# Supplementary material for: Neutrophils-to-Lymphocyte Ratio Is Associated with Progression and Overall Survival in Amyotrophic Lateral Sclerosis
Source: Biomedicines. 2022 Feb 1;10(2):354. doi: 10.3390/biomedicines10020354 (PMC8962424; doi:10.3390/biomedicines10020354)
Supplement: Supplementary file 1 [file biomedicines-10-00354-s001.zip › biomedicines-1511317-supplementary.pdf]

Supplemental Figure S1. Patients' disposition flow diagram in the PRO-ACT database (i.e. selection of the validation cohort)

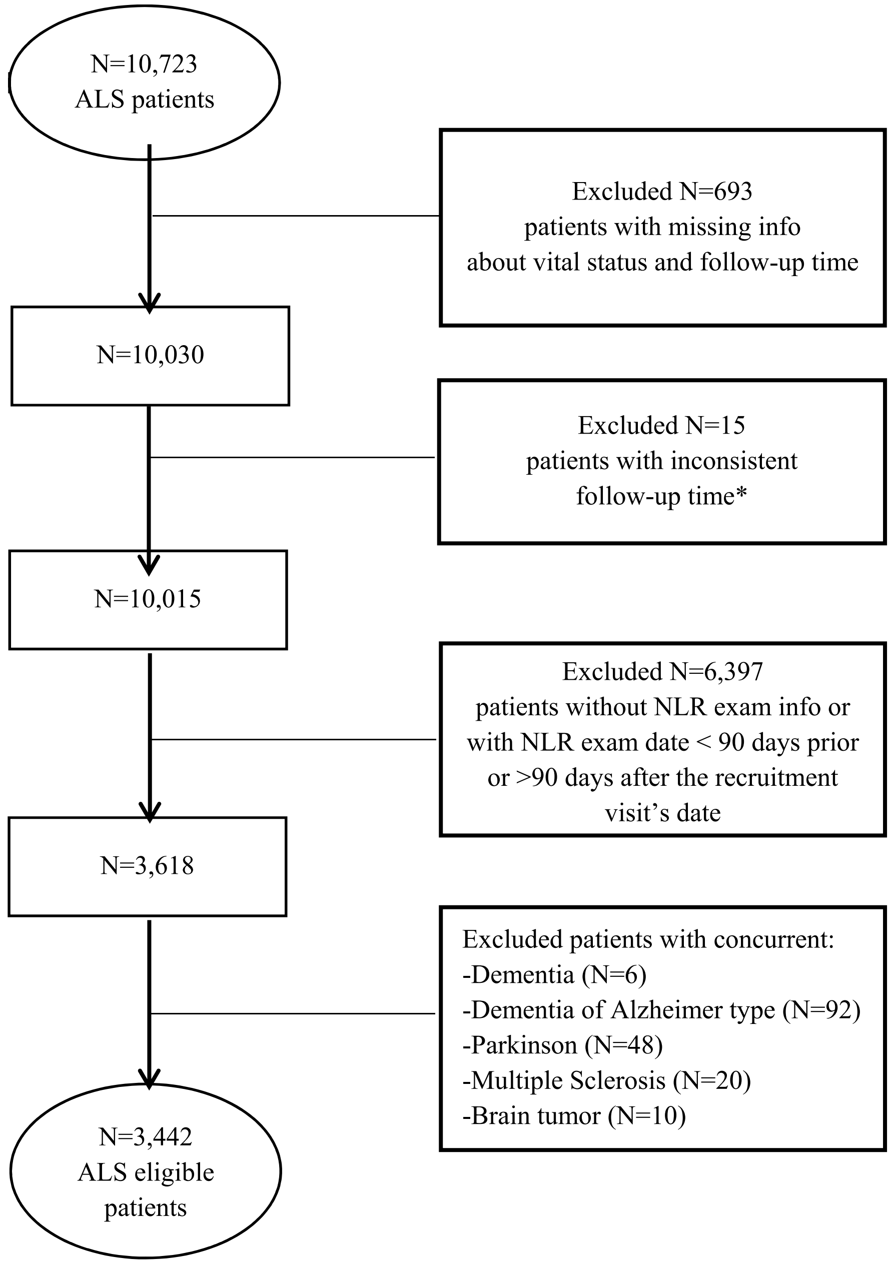

*There were 15 deceased patients with time to death interval (i.e. from recruitment visit to death dates) lower than the time to the last measurement (i.e. from the recruitment visit to the last date recorded from all databases).

Supplemental Table S1. Clinical characteristics of patients collected in the PRO-ACT database, overall and according to the tertiles of Neutrophil-to-Lymphocyte Ratio (NLR) distribution

**Variable**	**Category**	**All (N=3,442)**	**I: NLR < 1.519 (N=347)**	**II: NLR [1.519-2.326] (N=1,070)**	**III: NLR > 2.326 (N=2,025)**	**p-value**	**SMD**	**Missing data (%)**
Gender - N(%)	Females<br>Males	1302 (37.8)<br>2140 (62.2)	147 (42.4)<br>200 (57.6)	443 (41.4)<br>627 (58.6)	712 (35.2)<br>1313 (64.8)	0.001	0.099	0%
Age at recruitment (years)	Mean±SD	55.50±11.36	53.74±11.90	54.68±11.20	56.24±11.28	<0.001	0.145	19.4%
Age at diagnosis (years)	Mean±SD	54.23±11.47	52.15±11.74	53.66±11.46	54.86±11.38	0.022	0.157	63.8%
Age at onset (years)	Mean±SD	53.70±11.39	51.89±11.97	52.87±11.26	54.46±11.29	<0.001	0.149	19.6%
Disease duration (months)§	Median [IQR]	18.02 [11.50-28.02]	18.37 [11.93-28.09]	18.40 [11.47-28.65]	17.71 [11.43-27.70]	0.512*	0.042	19.6%
Disease duration§ – N(%)	≤ 12 months<br>13-24 months<br>25-36 months<br>37-48 months<br>> 48 months	754 (27.2)<br>1103 (39.8)<br>551 (19.9)<br>200 (7.2)<br>160 (5.8)	75 (26.1)<br>117 (40.8)<br>47 (16.4)<br>29 (10.1)<br>19 (6.6)	236 (27.5)<br>334 (39.0)<br>175 (20.4)<br>59 (6.9)<br>53 (6.2)	443 (27.3)<br>652 (40.1)<br>329 (20.3)<br>112 (6.9)<br>88 (5.4)	0.530	0.114	19.6%
Site of onset - N(%)	Bulbar<br>Limb<br>Limb and bulbar	713 (20.8)<br>2693 (78.4)<br>29 (0.8)	84 (24.2)<br>261 (75.2)<br>2 (0.6)	233 (21.8)<br>828 (77.5)<br>7 (0.7)	396 (19.6)<br>1604 (79.4)<br>20 (1.0)	0.204	0.080	20.0%
FVC – N(%)	<80%<br>≥80%	461 (44.9)<br>565 (55.1)	44 (44.9)<br>54 (55.1)	129 (40.7)<br>188 (59.3)	288 (47.1)<br>323 (52.9)	0.174	0.087	70.2%
Use of Riluzole at screening - N(%)	No<br>Yes	956 (32.0)<br>2031 (68.0)	114 (38.4)<br>183 (61.6)	332 (35.7)<br>598 (64.3)	510 (29.0)<br>1250 (71.0)	<0.001	0.133	13.2%
ALSFRS-R	Mean±SD	37.17±5.57	36.83±6.26	37.51±5.51	37.05±5.49	0.346	0.080	65.4%
Time from recruitment to last follow-up (years)§	Median [IQR]	1.08 [0.79-1.51]	1.08 [0.84-1.51]	1.15 [0.83-1.52]	1.07 [0.76-1.51]	0.019*	0.086*	0%
Mortality rate§	events/PYs (rate per 100 PYs)	1020/3889 (26.2)	89/395 (22.6)	292/1258 (23.2)	639/2236 (28.6)	0.004°	---	

§disease duration is defined as the time interval occurred from ALS onset to trial's recruitment

p-values from ANOVA models or Chi-Square statistics for continuous and categorical variables, respectively. # p-values from Fisher exact test; ° p-value from Poisson regression; *analysis on log-transformed values. NLR: Neutrophil-to-Lymphocyte Ratio; SD: standard deviation; IQR: interquartile range (i.e. first-third quartiles); SMD: standardized mean difference (i.e. the average of all possible standardized mean differences); PYs: Person-Years

Supplemental Figure S2. Kaplan-Meier (KM) survival curves according to NLR tertiles (panel **A**) and tree-based cut-off (panel **B**) of patients collected in the PRO-ACT database. Censored observations are evidenced on the KM curves as tick marks ("+")

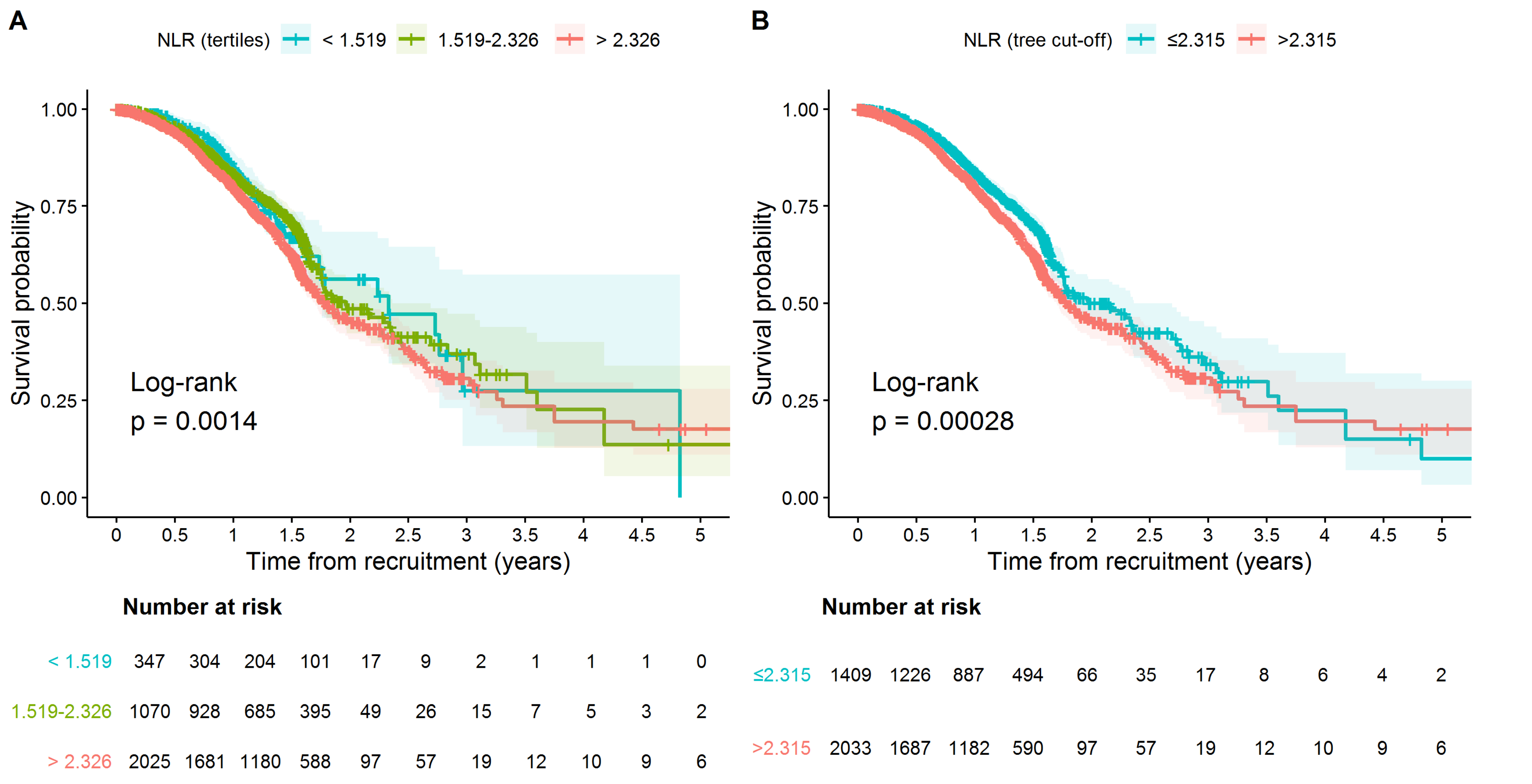

Supplemental Table S2. Association between Neutrophil-to-Lymphocyte Ratio (NLR) values and mortality rate. Results from Cox univariable and multivariable models.

Model type	Variables included into the model (covariates)	Covariates type	Groups (NLR cutoffs) comparison	HR (95%CI)	p-value	Test for functional form	Test for PH (p-value)[#]

						**(p-value)**[#]	
Univariable (unadjusted)	NLR	Continuous	--	1.34 (1.16-1.54)	<0.001	0.068	0.848
	NLR (tertiles)	Categorical	[1.519-2.326] vs. < 1.519	0.99 (0.78-1.25)	0.905	--	0.555
			> 2.326 vs. < 1.519	1.25 (1.00-1.56)	0.049	--	0.465
	NLR (tree-based cut-off)	Categorical	≤2.315 vs. >2.315	1.27 (1.11-1.44)	<0.001	--	0.450
Multivariable (adjusted)	NLR	Continuous	--	1.15 (0.98-1.36)	0.089	0.137	0.818
	Age at recruitment (years)	Continuous	--	1.05 (1.04-1.06)	<0.001	0.023	0.614
	NLR (tertiles)	Categorical	[1.519-2.326] vs. < 1.519	0.95 (0.73-1.25)	0.717	--	0.771
			> 2.326 vs. < 1.519	1.12 (0.87-1.44)	0.381	--	0.856
	Age at recruitment (years)	Continuous	--	1.05 (1.04-1.05)	<0.001	0.020	0.605
	NLR (tree-based cut-off)	Categorical	≤2.315 vs. >2.315	1.17 (1.01-1.35)	0.042	--	0.804
	Age at recruitment (years)	Continuous	--	1.05 (1.04-1.05)	<0.001	0.022	0.606

HR: Hazard Ratio; CI: Confidence Interval; PH: Proportional Hazards

[#]To check the adequacy of the fitted Cox regression models (i.e. checking of the functional form of a continuous covariate included into the Cox model and the assessment of the PH assumption), the Kolmogorov-type supremum test for functional form and for PH assumption was performed on the basis of 1,000 data replicates (simulations). As no polynomial (and interaction) terms of each continuous covariate were included into each Cox model, the test for functional form assessed whether a linear relationship existed between a one unit increase of the continuous coviariate and the risk of death.
